# Damage Diagnosis Framework for Composite Structures Based on Multi-Dimensional Signal Feature Space and Neural Network

**DOI:** 10.3390/ma18163834

**Published:** 2025-08-15

**Authors:** Jian Wang, Jing Wang, Shaodong Zhang, Qin Yuan, Minhua Lu, Qiang Wang

**Affiliations:** 1 China Comservice Wangying Technology Co., Ltd., Nanjing 210000, China; wangjian.jswy@chinaccs.cn (J.W.); yuanqin.jswy@chinaccs.cn (Q.Y.); luminhua.jswy@chinaccs.cn (M.L.); 2 School of Electronic Information, Nanjing Vocational College of Information Technology, Nanjing 210023, China; 3 College of Automation and College of Artificial Intelligence, Nanjing University of Posts and Telecommunications, Nanjing 210023, China; 1218053409@njupt.edu.cn (S.Z.); wangqiang@njupt.edu.cn (Q.W.)

**Keywords:** composite, structural health monitoring (SHM), Lamb waves, multi-scale analysis, damage assessment

## Abstract

It is particularly important to ensure the safety of engineering structures, such as aerospace vehicles and wind turbines, most of which are made of composite materials. A sudden failure of the structure may happen following the accumulation of structural damage. Since they are sensitive to tiny damage and can propagate through engineering structures over a long distance, Lamb waves have been widely explored to develop highly efficient damage detection theories and methodologies. During propagation, affected by the mechanical properties of the structure, a large amount of information and features related to structural states can be reflected and transmitted by Lamb waves, including the occurrence and extent of structural damage. By analyzing the effect of damage acting on Lamb waves, a multi-scale wavelet transform analysis is adopted to extract multi-feature parameters in the time–frequency domain of the acquired signals. With the help of the nonlinear mapping ability of a neural network, a damage assessment model for composite structures is constructed to realize the evaluation of typical structural damage at different levels. The results of an experiment conducted on an epoxy–glass-fiber-reinforced plate show that the extracted multi-feature parameters of Lamb waves in the time–frequency domain are sensitive to the accumulated typical damage. The damage assessment model can properly evaluate the damage degree with satisfactory accuracy.

## 1. Introduction

At present, alloy materials are gradually being replaced by composite materials because of composite materials’ advantages of high strength, light weight, high specific modulus, and strong corrosion resistance. Composite materials are widely used in various fields, including aerospace, wind power, automotive, and civil engineering. However, structural damage may develop from the original defects generated in the manufacturing process of composite materials [1]. In addition, the composite structure will also suffer from a variety of damage types under impact and pressure during its service time. The structural properties will consequently change depending on the severity of damage. Damage degree assessment has been an important issue in structural health monitoring for composite structures.

In existing SHM research, methods based on structural modal parameters, such as natural frequency and frequency response functions, have limited influence on the overall parameters of the structure in the early stage [2,3]. So, it is difficult to find distinguishable changes when the damage is still in an early stage of development [4]. Damage identification based on electromechanical impedance (EMI) is also a hotspot of research, since this method is sensitive to tiny damage [5]. However, it depends heavily on the constructed model, which is time-consuming and inaccurate. Meanwhile, sensors may be easily contaminated by the ambient environment, i.e., temperature and humidity.

Guided-wave-based SHM methods extract the signal differences between a baseline signal and a monitoring signal to assess the damage, in combination with advanced signal processing techniques. They are considered some of the most promising monitoring methods because of their sensitivity to small damage and their large monitoring area with only a few sensors [6,7,8,9]. However, the response of Lamb waves can be easily modulated by various factors, such as the type of damage and the properties of the structure [10]. The damage in the structure will cause changes in the inherent parameters and have different degrees of influence on the mechanical properties of the structure [11].

However, with the existing guided-wave-based SHM methods, which are too complicated and prone to being influenced by many factors, damage assessment and monitoring studies are impractical. The formation and development of structural damage are affected by many factors, especially for composite structures, which are complex, with diverse structures and different properties [12,13,14]. Due to the limitation of available information, it is difficult to reflect the process of change in the structural state based on traditional methods like damage scattering [15].

In this research study, the wavelet packet analysis method, which has been researched by the authors, is used to analyze the damage signal in the time domain and frequency domain [16]. Time-domain characteristic parameters, i.e., waveform features (WFs) and waveform peak features (WPs), and frequency-domain characteristic parameters, i.e., energy distribution (ED) and energy percentage (E), are extracted. The damage feature vector is established, and the damage information standard database is constructed. A customized distance is proposed to describe the migration of feature vectors, which could reduce the interference from the material uniformity and sensor inconsistency. Furthermore, with the help of the nonlinear mapping ability of neural networks, a BP (backpropagation) neural network is combined with a genetic algorithm (GA), which improves the accuracy of damage degree evaluation in composite structures. At last, this method is verified on a carbon fiber composite plate with different degrees of hole damage and crack damage. The experiments show the effectiveness of the proposed method.

## 2. Fundamental Analysis and Overview

Lamb waves are the elastic waves formed by the coupling of a transverse wave and a longitudinal wave in a structure with two parallel surfaces. Because the propagation process of Lamb waves is very complex, it is easily affected by its own parameters and structural properties, such as frequency and plate thickness. According to the particle–phase relationship in single-layer isotropic materials, Lamb waves can be divided into symmetric waves and antisymmetric waves, and each wave has multiple modes. The propagation characteristics can be explained by Formula (1) [17]:

(1) $$\frac{tan\beta d}{tan\alpha d}=-{\left[\frac{4\alpha \beta k^{2}}{{\left(k^{2}-\beta^{2}\right)}^{2}}\right]}^{\pm 1}$$

where $\alpha^{2}=\left(\omega^{2}/c_{1}^{2}\right)-k^{2}$ and $\beta^{2}=\left(\omega^{2}/c_{2}^{2}\right)-k^{2}$. In addition, *d* is the thickness of the plate, and ω is the angular frequency. $c_{1}$ is the transverse wave velocity, and $c_{2}$ is the longitudinal wave velocity. *k* is the wave number, calculated as $k=\omega /c_{3}$, where $c_{3}$ is the phase velocity of the Lamb wave. At last, the superscript positive sign corresponds to symmetric mode S, and the negative sign corresponds to antisymmetric mode A.

Therefore, according to the above analysis, there are dispersion and multimodal effects on the propagation of Lamb waves. The Lamb wave signal excited by a broadband excitation signal has many modes, and the propagation speed of the same mode at different frequencies is also different. At this time, the signal cannot be analyzed clearly. Therefore, in the study of active Lamb wave health monitoring, the expression of shear stress in the wave number domain can be obtained as follows [16]:

(2) $${\left.u\left(x,\;t\right)\right|}_{y=d}=-\frac{a\tau_{0}}{Gs}\sum_{k^{s}}sin\left(k^{s}a\right)\frac{C_{s}^{\tau }\left(k^{s}\right)}{B_{s}\left(k^{s}\right)}e^{-i\left(k^{s}x-\omega t\right)}-\frac{a\tau_{0}}{Gs}\sum_{k^{A}}sin\left(k^{A}a\right)\frac{C_{s}^{\tau }\left(k^{A}\right)}{B_{A}\left(k^{s}\right)}e^{-i\left(k^{A}x-\omega t\right)}$$

The formula contains many parameters: *x* is the distance, *Gs* is the shear modulus, *τ* is shear stress, *k* is the wave number, *sin*(*ka*) is the amplitude function, *e*^−*i*(*kx*−*wt*)^ is harmonic function, *A* and *S* are the modes, and *C* and *B* are the functions of *k*. For composite structures, anisotropic properties introduce many interesting but somewhat complex phenomena in Lamb wave propagation, such as direction-dependent speed, and difference between phase and group velocities.

Above all, each parameter will change due to the damage initiation. According to the above formula, the influence of damage on Lamb waves is not only unilateral but also affected and changed in the time domain, frequency domain, and time–frequency domain. Therefore, it is necessary to analyze the influence of damage information on these parameters.

**Figure 1:**
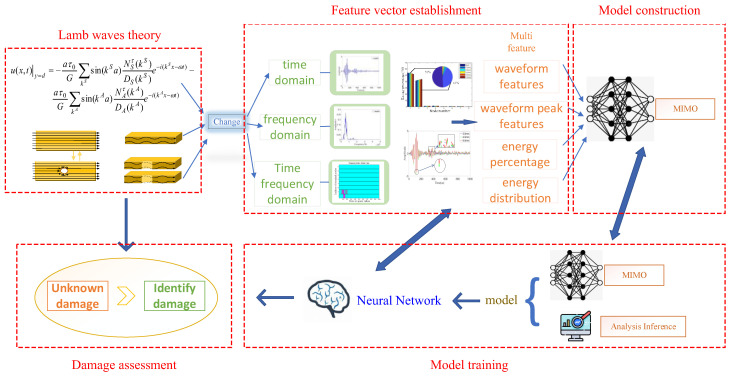
Overall concept map.

Through the analysis of Lamb wave propagation process under piezoelectric excitation, starting from the signal propagation equation and the time–frequency domain of the signal, using the method of signal processing, the multimodal response of Lamb waves to structural changes and different types and degrees of damage is used to analyze and extract the multi-characteristics of the signal. Starting from the Lamb wave excitation equation based on piezoelectric sensors, this paper analyzes the “changed” parts and then analyzes how these “changed” parts behave in the actual response and the changes in the response signal caused by damage. Then, the relevant time–frequency-domain multi-features are extracted through the outdated frequency-domain analysis method, and damage diagnosis, evaluation, and prediction are realized with the help of a machine learning method. The main research and analysis steps are shown in Figure 1.

The damage mechanism of composite materials is very complicated, and it is difficult to find a clear functional relationship based on inherent system parameters such as natural frequency and frequency response function. Moreover, the period of damage occurrence and the recognition effect will also produce poor results under the influence of many factors. The neural network can automatically learn the damage samples extracted by the system and can approach the nonlinear mapping caused by the damage with high accuracy. Therefore, this advantage can be utilized in the evaluation of the damage degree. In this paper, a combination of a neural network and an intelligent algorithm is used to evaluate the different degrees of damage in composite materials. Also, the GA is adopted to achieve the optimization of a BP neural network to predict the damage degree.

Therefore, a multi-dimensional signal feature space- and neural network-based damage monitoring and assessment method is designed as shown in Figure 1. In the proposed method, based on the mechanism of the occurrence and development of damage in composite structures based on Lamb wave response, multiple time–frequency-domain features are analyzed and extracted. And a multi-input multi-output neural network is adopted to construct a damage monitoring and evaluation model. The main advantage of this framework design is to comprehensively utilize the characteristics of the response signal waveform and time–frequency-domain energy before and after damage to identify the occurrence and degree of damage, avoiding the signal analysis difficulties caused by complex propagation characteristics.

## 3. Multi-Dimensional Signal Feature Space-Based Damage Monitoring and Assessment

### 3.1. Analysis of Lamb Wave Structural Response

**Figure 2:**
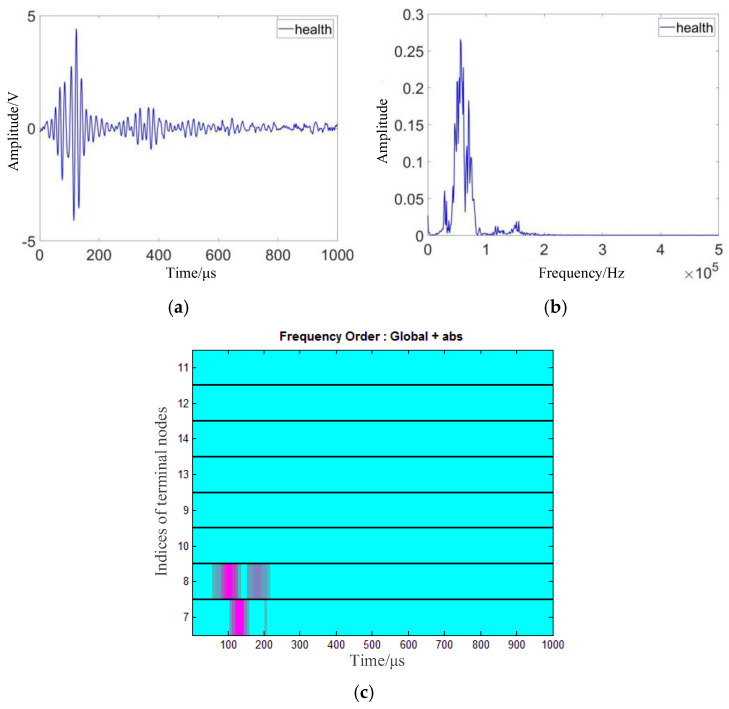
Structural response signal of Lamb wave under lossless condition. (**a**) Time-domain waveform; (**b**) frequency spectrum; (**c**) time-frequency diagram.

According to the above analysis, it is necessary to acquire typical damage signals for detailed signal analysis to find out which are the local changes in the formula, so that the characteristic parameters can be put forward according to these changes. The damage forms in composites are various, including fracture, delamination, crack and through-hole. Different damage forms have unique characteristics. Since the interaction mechanism between structural damage and Lamb waves is complex, it is difficult to identify the damage type from acquired signals in the time domain and frequency domain and single characteristic parameters [18,19]. Through-hole damage is a typical form in composite structures caused by external action. For convenience, this kind of damage is selected as the typical damage type for further experiments to explore the interaction mechanism mentioned above. On an epoxy–glass-fiber-reinforced plate, two piezoelectric wafers are used as the exciter to generate Lamb waves and the receiver to receive the response signal. The time-domain and frequency-domain waveforms of structural response signals under non-destructive conditions are shown in Figure 2a,b. After wavelet packet transform, the time–frequency diagram is obtained as shown in Figure 2c. As can be seen from the figure, the colors of band 7 and band 8 are the strongest, which indicates that the signal frequency is mainly concentrated between these two bands (i.e., between 0–62.5kHz and 62.5–125kHz). There is no distribution in other frequency bands.

**Figure 3:**
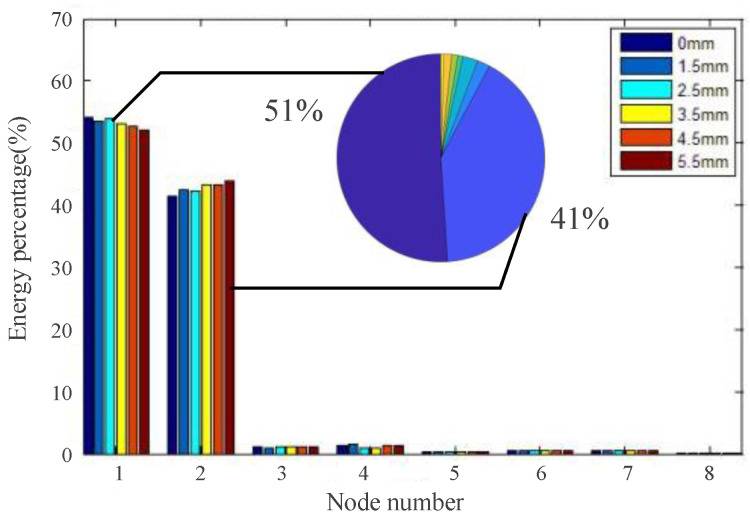
Comparison of energy distribution of different damage degrees (through-hole).

In the same way, the time–frequency diagrams of structural response signals for different degrees of damage are analyzed. By analyzing the main frequency distribution of the acquired signals, the energy concentration degrees of the third layer of different degrees of damage are compared, and the energy distribution diagram is shown in Figure 3. As can be seen from the result, the energy is mainly concentrated in the first and second nodes of the third layer.

According to the analysis results shown in Figure 2 and Figure 3, the signal is transformed into the time–frequency domain. Resulting from the changes in the signal energy and the wavelet packet coefficient caused by damage, the waveform feature, waveform peak feature, energy percentage, and energy distribution are extracted. Then, the occurrence and development of damage can be further analyzed in multiple dimensions [20].

### 3.2. Feature Extraction

Wavelet analysis uses a wavelet basis function to approximately determine a certain signal or function by expansion and translation, while wavelet packets can overcome the lack of wavelet decomposition capability when analyzing high-frequency signals. The decomposition process of wavelet packets is the process of bandpass filtering, in fact [21]. According to the wavelet packet algorithm which is illustrated as Formulas (3) and (4), the decomposition process of a signal is actually the result of the convolution of wavelet packet coefficients *P* and bandpass filters *H* and *G*. Then, sampling analyses are conducted at intervals. The data are halved when each layer of signal is decomposed. The high- and low-frequency parts of the damage signal are decomposed, and the decomposed damage signal can be divided into different frequency bands. Finally, the feature information of each frequency band extracted by wavelet packet analysis can be used to evaluate the damage degree.

(3) $$\left\{\begin{array}{c} p_{0}^{l}(t)=f(t),\\ p_{l}^{2j-1}=\sum_{k}H(k-2t)p_{l-1}^{j}(t),\\ p_{l}^{2j}=\sum_{k}G(k-2t)p_{l-1}^{j}(t),\\ t=1,2,\ldots ,2^{l-1};j=1,2,\ldots ,2^{l};L=\log_{2}N. \end{array}\right.$$

(4) $$p_{l}^{j}(t)=2\left[\sum_{k}h(t-2k)p_{l+1}^{2j-1}+\sum_{k}g(t-2k)p_{l+1}^{2j}\right]$$

where $p_{l}^{j}$ is the *j*-th wavelet packet coefficient of the *l*-th layer, *l* is the number of decomposition layers, *n* is the signal length, *G* and *H* are the wavelet packet decomposition filters, and *g* and *h* are the wavelet packet reconstruction filters.

A Lamb wave is a type of non-stationary signal. The wavelet packet analysis in the time–frequency-domain analysis method, which can overcome the shortcoming of performing a conversion from the time domain into the frequency domain, can be adopted to deal with Lamb wave signals. At the same time, this method is sensitive to damage generating in early stages (i.e., tiny damage which is undetectable for most of current technologies), and the wavelet packet method can improve the accuracy of damage assessment.

According to the analysis in the previous section, wavelet packet decomposition is used to extract the waveform features, waveform peak features, energy percentage, and energy distribution. The formulas are as follows:

(5) $$Wf(l,j)=\frac{\sqrt{\frac{1}{n}\sum_{i=1}^{N}{\left[p_{l}^{j}(i)\right]}^{2}}}{\frac{1}{N}\sum_{i=1}^{N}p_{l}^{j}(i)}$$

(6) $$Wp(l,j)=\frac{\max\{p_{l}^{j}(i)\left|i=0,1,\ldots ,2^{l}-1\right.\}}{\sqrt{\frac{1}{N}\sum_{i=1}^{N}{\left[p_{l}^{j}(i)\right]}^{2}}}$$

(7) $$Ed(l,j)=\frac{1}{N}\sum_{i=1}^{j}{\left[p_{l}^{j}(i)-\frac{1}{N}\sum_{i=1}^{N}p_{l}^{j}(i)\right]}^{2}$$

(8) $$E(l,j)=\frac{\frac{1}{N}\sum_{i=1}^{N}{\left[p_{l}^{j}(i)\right]}^{2}}{\sum_{i=1}^{j}\left(\frac{1}{N}\sum_{i=1}^{N}{\left[p_{l}^{j}(i)\right]}^{2}\right)}$$

*Wf* reflects the time-domain waveform characteristics of the reconstructed signal of node *j*, *Wp* reflects the peak characteristics of the reconstructed signal, *Ed* reflects the frequency-domain energy distribution of the *j*-th node signal, *E* reflects the percentage of the frequency-domain energy of the *j*-th node signal in the total energy of the *l*-th layer signal, and *N* is the length of the coefficient.

To use these formulas to reflect the different characteristics of damage, the wavelet basis function must be firstly determined, and the establishment of the wavelet basis function is mainly based on its compactness, regularity, vanishing distance, symmetry, and support length. A Lamb wave signal is a type of non-stationary signal. It has transient and complex characteristics. In order to obtain its local characteristics, the compact support in the time domain should be considered as much as possible, and a certain energy analysis ability in the frequency domain should be ensured. Therefore, wavelet basis functions with fast frequency band and slow speed attenuation are chosen. A wavelet basis with good regularity can accurately describe the energy characteristics of a signal better. A wavelet basis with good symmetry can reduce the edge distortion of the signal, and appropriate vanishing moments can suppress the high-frequency noise of the signal. Combined with the above analysis, the DB4 wavelet basis function is selected.

Therefore, the collected signal is decomposed into three layers by using the DB4 wavelet basis function, and the third layer is divided into eight frequency bands, including high-frequency and low-frequency information of the signal. Combined with the proposed features, the damage characteristics can be well reflected.

### 3.3. Separability Comparison of Feature Parameters

In the evaluation of the damage degree, the proper selection of characteristic parameters can improve the accuracy of evaluation [18]. In order to obtain the features that reflect the essence of damage analysis, the features that have a greater degree of differentiation are selected, and the other features that have a smaller impact on classification are discarded. In this paper, the Euclidean distance (determined by Formula (9)) is selected as the assessment method for features. The smaller the Euclidean distance is, the more similar the features are. On this basis, the intra-class distance (determined by Formula (10)) and inter-class distance (determined by Formula (11)) of damage features are defined as the assessment basis.

(9) $$D^{2}(X_{i},X_{j})=\sum_{k=1}^{n}{\left(X_{ik}-X_{jk}\right)}^{2}$$

(10) $$\bar{D_{m}^{2}(\{X_{i}\},\{X_{j}\})}=\frac{1}{N(N-1)}\sum_{i=1}^{N}\sum_{\begin{array}{l} j=1\\ i\neq j \end{array}}^{N}\sum_{k=1}^{n}{\left(x_{ik}-x_{jk}\right)}^{2}$$

(11) $$\bar{D_{n}^{2}(\{X_{i}\},\{X_{j}\})}=\frac{1}{N_{x}N_{y}}\sum_{i=1}^{N_{x}}\sum_{j=1}^{N_{y}}\sum_{k=1}^{n}{\left(x_{ik}-y_{jk}\right)}^{2}$$

For the set of eigenvectors in the same degree of damage, the intra-class distance can represent the dispersion degree of eigenvectors in the set. The smaller the value is, the closer it is. The inter-class distance represents the difference of characteristic vectors of different degrees of damage. When the intra-class distance of any damage feature vector is less than the inter-class distance of other damage classes, it can be considered that the damage feature vector has good separability. On this basis, the separability criterion of damage degree is proposed (Formula (12)). The smaller the criterion is, the better the separability of the damage degree of *X* and *Y* is.

(12) $$J_{X,Y}=\frac{D^{2}(\{X_{i}\},\{Y_{j}\})}{\bar{D^{2}(\{X_{i}\},\{X_{j}\})}+\bar{D^{2}(\{Y_{i}\},\{Y_{j}\})}}$$

### 3.4. Assessment of Damage Degree

After the collected signal is studied by wavelet multiresolution analysis, the multi-scale feature parameters are extracted, including waveform feature *Wf*, waveform peak *Wp*, energy distribution *Ed*, and energy percentage *E*. Later, the damage information standard library is established according to these parameters. Then, a neural network is used for damage identification as shown in Figure 1. In this paper, a typical BP neural network and its improved version, the GA-BP neural network, are adopted to build the damage assessment model. The multi-dimensional signal feature samples are divided into a training part and a test part. Then the networks are trained, and the unknown damage can be tested to validate the method.

## 4. Experimental Verification and Result Analysis

### 4.1. Design of Damage Assessment System

**Figure 4:**
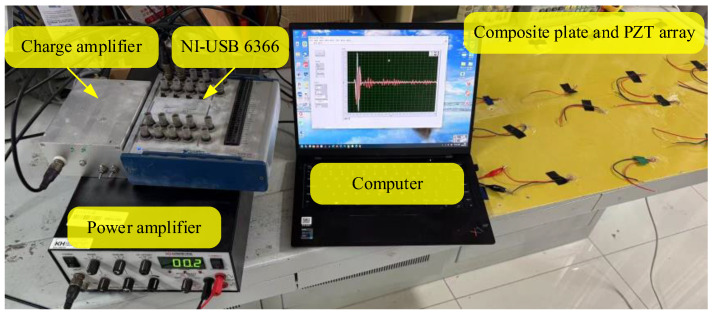
Experimental setup.

In this paper, the damage assessment system is composed of three modules, a damage signal monitoring module, a damage signal analysis and processing module, and a damage degree assessment module, as shown in Figure 1. The damage signal monitoring module is mainly composed of data acquisition equipment, a power amplifier, a charge amplifier, and the structure to be tested, as shown in Figure 4. This module uses on-line monitoring technology, designs the sensor array according to the physical condition of the structure, and excites the Lamb wave signal through the computer [22]. Because the amplitude of the excitation signal of the upper computer is limited and the Lamb wave propagation signal in the structure will be attenuated, it is necessary to use the power amplifier to amplify the excitation signal. The amplitude of the signal received by the piezoelectric sensor is about 25 mV. This signal is amplified and filtered by the charge amplifier. Finally, the computer is used to store the received signals.

In the damage signal analysis and processing module, the time–frequency-domain analysis method is used to analyze the damage signal. According to the change in the characteristic parameters in the time–frequency domain (including the wave feature *Wf* and wave peak *Wp* in the time domain and the energy distribution *Ed* and energy percentage *E* in the frequency domain), there parameters are extracted as the characteristic vectors of damage, on the basis that a damage information standard database is established.

The damage degree evaluation module is realized through the neural network. The damage sample set is selected from the damage information standard database, which is divided into a training set and a test set. Then the neural network is trained and tested. Finally, unknown damage is used to verify the evaluation accuracy of the network.

### 4.2. Design of Experimental Scheme

The material used in the experiment was an epoxy–glass-fiber-reinforced plate, whose size was 1000 mm × 500 mm × 3 mm (the density was 1960 kg/m^3^, Young’s modulus was 20 GPa, and Poisson’s coefficient was 0.17). Based on the Lamb wave propagation dispersion characteristics, in order to improve signal resolution and obtain a response signal which is simple and easy to identify, a narrow-band excitation signal was selected by considering the time resolution and frequency resolution. The center frequency was limited in the range of 20 kHz~200 kHz, and only A0 or S0 mode was excited. After analysis and experimental testing, the center frequency was selected as 60 kHz. The sampling rate was set to be 1 MSPS, and the sampling length was 1000.

**Figure 5:**
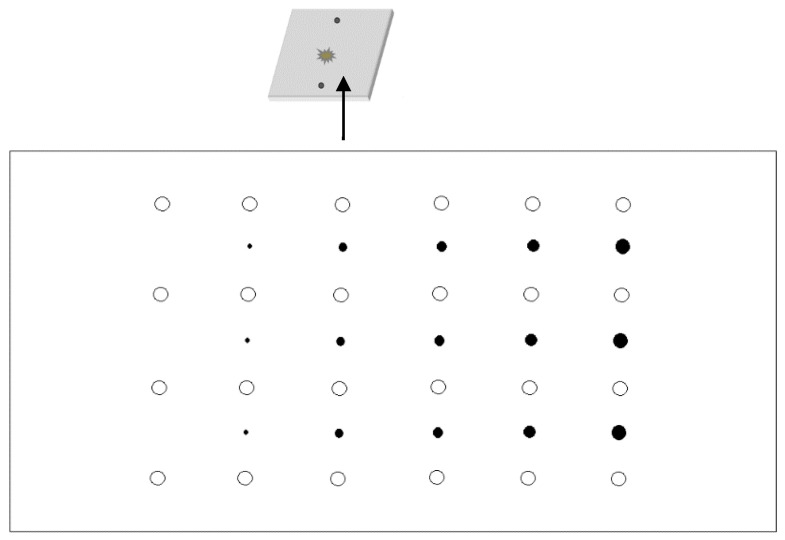
Multiple linear array layout.

The aim of this experiment is to collect data on different degrees of damage. To reduce the large number of received data induced by the changeable damage levels, a single linear array was used as the experimental sensor array. In order to obtain and analyze as many experimental data samples as possible, the sensor array with 24 sensors was designed to have four rows and six columns, as shown in Figure 5. In this experiment, PZT-5A piezoelectric elements, whose diameter and thickness were 8 mm and 0.48 mm, respectively, were utilized.

Different degrees of damage were induced in the composite plate. Hole damages of sizes of 0 mm, 1.5 mm, 2.5 mm, 3.5 mm, 4.5 mm, and 5.5 mm were induced in the longitudinal direction. Crack damage to three different degrees, which were non-destructive, and 20% and 50% of the structure thickness, was induced in the transverse direction.

In the longitudinal direction, through-hole damage was induced to six different degrees. During the experiment, each sensor element was successively actuated to generate Lamb waves, and all its adjacent elements received Lamb waves. Thus, 81 groups of signals were obtained once, and 486 groups of signals were obtained for six different degrees of hole damage. In the transverse direction, crack damage was induced, divided into three degrees, and 252 groups of signals were collected.

### 4.3. Analysis of Experimental Results

**Figure 6:**
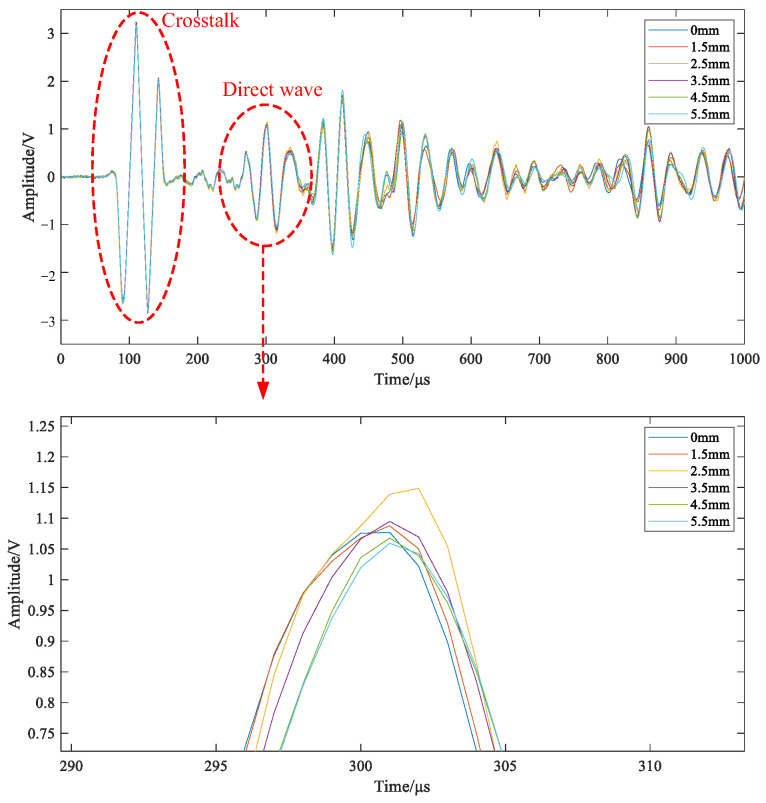
Signals corresponding to hole damage of different sizes.

The feature parameters extracted from the acquired signals a have certain physical meaning, with different dimensions. Therefore, it is necessary to normalize the extracted feature parameters to the scale. After normalization, the separability of time-domain eigenvectors, frequency-domain eigenvectors, and time–frequency-domain eigenvectors of typical damage through-holes (0 mm, 1.5 mm, 2.5 mm, 3.5 mm, 4.5 mm, and 5.5 mm) is compared, as shown in Figure 6.

According to the above calculated parameters (including waveform feature *Wf*, waveform peak *Wp*, energy distribution *Ed*, and energy percentage *E*), the three feature vectors are defined as follows:

Eigenvectors in the time domain: [WF0, WF1, …, WF7, WP0,WP1, …,WP7];

Eigenvectors in the frequency domain: [ED0, ED1, …, ED7, E0, E1, …, E7];

Eigenvectors in the time–frequency domain: [WF0, WF1, …, WF7, WP0,WP1, …, WP7, ED0, ED1, …, ED7, E0, E1, …, E7].

**Figure 7:**
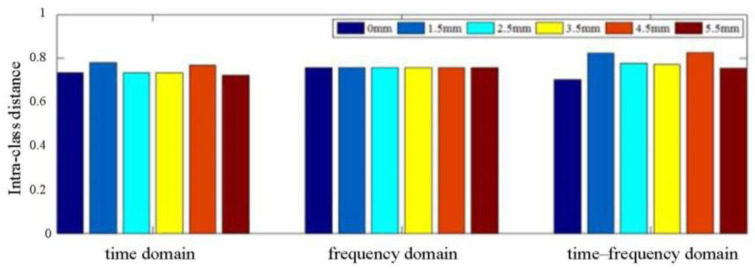
Comparison of intra-class distance of feature vectors.

**Figure 8:**
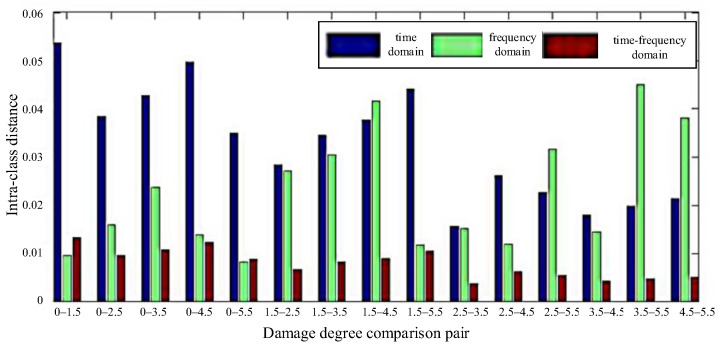
Comparison of distance between different damage feature vectors.

**Figure 9:**
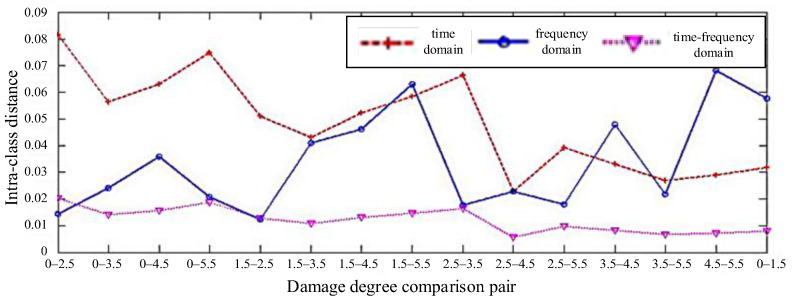
Comparison of separability of different damage feature vectors.

The statistics of the intra-class distance of the different degrees of damage eigenvectors are shown in Figure 7. It can be seen that the eigenvectors of damage of different degree have great differences in the time–frequency domain. Their mean value and mean square deviation were calculated in the time domain (0.7466/0.0229), frequency domain (0.7579/0.0046), and time–frequency domain (0.7759/0.0462), respectively. It can be observed that the mean value and mean square deviation in the time–frequency domain are greater than those in the time–frequency domain, indicating that the separability of the time–frequency domain can be used to identify damage of different degree with higher accuracy. Figure 8 shows the inter-class distance statistics of different degree damage eigenvectors, and Figure 9 shows the separability comparison of different degree damage eigenvectors. From Figure 7, Figure 8 and Figure 9, it can be seen that the intra-class distances of the eigenvectors in the time–frequency domain are smaller than the inter-class distances in the time-frequency domain. At the same time, their mean square deviations were calculated (0.018 in the time domain, 0.013 in the frequency domain, and 0.004 in the time–frequency domain), and it was found that the mean square deviations are the smallest among them. This shows that the distribution of the extracted feature vectors is relatively even. The differences between the features are large, and the separability of different kinds of damage is great. However, by comparing the eigenvectors of two different degrees of damage, it can be found that their separability criteria fluctuate. The waveform of the damage signal is complex and diverse, and there are many factors affecting the characteristics of the received signals in the time domain. Therefore, the reliability and accuracy of damage assessment will be reduced by simply using the waveform eigenvectors in the time domain. In the frequency domain, the separability is volatile and unstable, so damage cannot be precisely assessed based on the data in the frequency domain.

In order to ensure the reliability and accuracy of damage degree assessment, the damage feature vector in the time–frequency domain is selected as the feature parameter for damage degree evaluation. Six kind of hole damage samples with different degrees are selected from the damage sample information database, and each sample has 80 groups.

Then the samples are divided into 16 groups of test samples and 64 groups of training samples. After training, when the number of hidden layer neurons reaches 30, and the training error and test error of the BP neural network are minimum. For the GA-BP neural network, the training error and test error of the GA-BP neural network are much smaller than those of the BP neural network. Therefore, considering the training error and test error, the structure of the neural network is 32-30-6. The corresponding activation functions, loss function, and optimizer are the Sigmoid function, mean square error, and gradient descent, respectively.

In the training of the neural network, the final results of the network are different due to the randomness of the test samples, training samples, and initial weights of the network. Thus, strong generalization ability is needed in the network. Therefore, in order to compare the accuracy and stability of the BP neural network and the GA-BP neural network, 10 rounds of training and testing are carried out according to the network structure mentioned above. Mean and mean square deviation are used to analyze the performance of the two networks. The mean value can well reflect the ability of a network to identify different degrees of damage. The smaller the mean square error is, the stronger the generalization ability of the network is.

**Figure 10:**
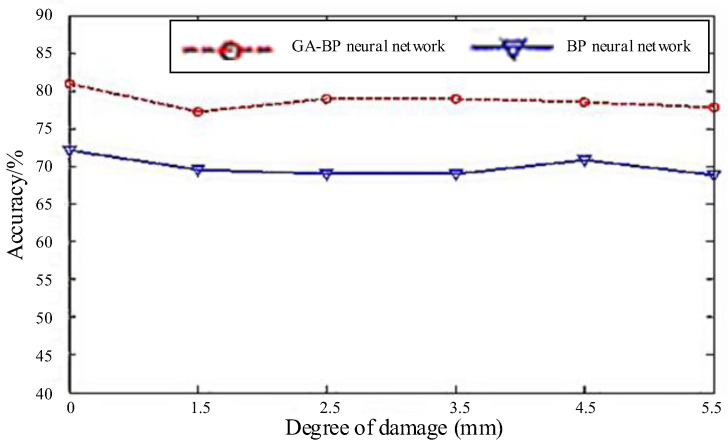
Comparison of hole damage evaluation accuracy between BP neural network and GA-BP neural network.

Conclusions can be further drawn from Table 1 and Figure 10. It can be seen from the mean value that the evaluation accuracy of the GA-BP neural network is higher than that of the BP neural network. The average accuracy rates of six different degrees of damage assessment in the GA-BP neural network are 80.9%, 77.3%, 78.9%, 78.9%, 78.5%, and 77.8%. The average correct rates of six kinds of damage in the BP neural network are 72.1%, 69.6%, 69%, 69%, 70.9%, and 68.9%, respectively. And the accuracy rates of the two neural networks are 78.85% and 68.9%. The results show that the evaluation accuracy of the network optimized by the genetic algorithm is significantly higher than that of the single BP neural network. From the process of training, we can find that the network only needs few training tests to achieve good recognition results.

The experimental results indicate that the test error of the GA-BP neural network is smaller than that of the BP neural network. However, in terms of mean square error, the generalization ability of the BP neural network is slightly higher than that of the GA-BP neural network. In general, using the GA-BP neural network to evaluate the damage degree can effectively improve the evaluation accuracy of the network, which reduces error in engineering applications.

In order to test the generalization ability of the system, crack damage is also selected to be evaluated. From the sample database, 80 groups of each damage degree sample set are selected, including 64 training sets and 16 test sets. The output node of the neural network is set to 3 because the evaluation of the crack damage degree is 3. After training, the number of hidden layer neurons is 28, and the structure of the whole network is 32-28-3.

Three different degrees (i.e., 0%, 20%, and 50%) of damage assessment accuracy can be obtained from Table 2. The accuracy rates of the BP neural network are 81.25%, 75%, and 75%. The accuracy rates of the GA-BP neural network are 87.5%, 87.5% and 93.5%, respectively. In terms of the accuracy of each degree of damage assessment, the accuracy rate of the GA-BP neural network is higher than that of the BP neural network. The accuracy of crack damage assessment is higher than that of hole damage assessment. The main reason is that the complexity of crack damage (three types) is lower than that of through-holes (six types), which consequently reduces the complexity of the network.

## 5. Conclusions

In this paper, time-domain characteristic parameters (WF and WP) and frequency-domain characteristic parameters (ED and E) are extracted to establish damage feature vectors. Then two kinds of neural networks, BP and GA-BP, are used to evaluate the damage degree. The average degree assessment of hole damage using the GA-BP neural network is 78.85%, and that of crack damage reaches up to 89.5%, which are higher than the accuracy rates of traditional the BP neural network. Experimental results show that the prediction of the damage degree can be improved to a certain extent via the multi-feature neural network evaluation method in this paper.

However, the method was carried out in a laboratory environment, and the damage type and position were not estimated. Further research is worth conducting to address damage type and position estimation under time-varying conditions.

## Figures and Tables

**Table 1:** Accuracy of identification of crack damage of different degree.

ANNT *	TE	Accuracy of Ten Tests						
		0	1.5	2.5	3.5	4.5	5.5	All
BPM	0.44	72.1	69.6	69	69	70.9	68.9	69.9
BPMSE	0.11	8.0	8.3	11.2	10.4	7.52	10.0	8.93
GABPM	0.06	80.9	77.3	78.9	79.7	78.5	77.8	78.8
GABPMSE	0.06	9.6	6.6	10.9	6.9	8.2	11.9	8.7

* ANNT: Artificial Neural Network type; TE: test error; BPM: BP neural network mean difference; BPMSE: BP neural network mean square error; GABPM: GA-BP neural network mean difference; GABPMSE: GA-BP neural network mean square error.

**Table 2:** Test accuracy of different neural networks.

Degree of Damage	BP			GA-BP		
	Test	Correct	Accuracy	Test	Correct	Accuracy
0%	16	13	81.3%	16	14	87.5%
20%	16	12	75%	16	14	87.5%
50%	16	12	75%	16	15	93.5%

BP: backpropagation; GA-BP: genetic algorithm backpropagation.

## Data Availability

The data presented in this study are available upon request from the corresponding author.
